# Serum osteocalcin levels are inversely associated with UACR in Chinese DKD patients: a meta-analysis of 20 clinical studies

**DOI:** 10.3389/fendo.2024.1514713

**Published:** 2024-12-02

**Authors:** Xiaolan Hu, Xiyu Wang, Chen Cai, Jiachen Guo, Xin Qian, Jiangyi Yu, Liji Huang, Shaofeng Xie

**Affiliations:** ^1^ Department of Endocrinology, Affiliated Hospital of Integrated Traditional Chinese and Western Medicine, Nanjing University of Chinese Medicine, Nanjing, China; ^2^ Department of Endocrinology, Affiliated Hospital of Nanjing University of Chinese Medicine, Nanjing, China

**Keywords:** osteocalcin, diabetic kidney disease, bone metabolism, case-control study, meta-analysis

## Abstract

**Objective:**

To systemically assess the relationship between serum osteocalcin levels and the progression of diabetic kidney disease (DKD) in the Chinese population.

**Methods:**

The PubMed, Web of Science, CNKI, Wanfang Database, VIP and Chinese Medical Journal full-text Database were searched. Two investigators independently reviewed the literature and extracted data based on predetermined inclusion and exclusion criteria. The Newcastle-Ottawa scale was used to assess the quality of the literature. The statistical analysis was performed using Stata16 software.

**Results:**

A total of 20 case-control studies encompassed 4 565 cases, consisting of 643 healthy controls (CN), 1 649 individuals with simple diabetes mellitus (DM), 1 305 with microalbuminuria (MI), and 968 with macroalbuminuria (MA). The meta-analysis results indicated that the serum osteocalcin levels in MI group were significantly lower than those in CN group and DM group [SMD = -1.15, 95% CI (-1.46, -0.85), P < 0.01; and SMD = -0.53, 95% CI (-0.69, -0.37), P < 0.01, respectively], and lower in the MA group compared to the CN group [SMD = -1.28, 95% CI (-1.79, -0.76), P < 0.01]. In the MA group, the serum osteocalcin levels were considerably lower compared to those in DM group and MI group [SMD = -0.93, 95% CI (-1.28, -0.58), P < 0.01; and SMD = -0.41, 95% CI (-0.65, -0.17), P < 0.01, respectively].

**Conclusion:**

The serum osteocalcin levels are typically reduced and show a negative correlation with the severity of proteinuria in Chinese patients with DKD. This indicates a decline in bone formation at early-stage in DKD patients, which worsens as the disease progresses.

**Systematic Review Registration:**

https://www.crd.york.ac.uk/PROSPERO/,identifier CRD42024580324.

## 1 Introduction

Diabetic kidney disease (DKD) is a primary microvascular complication affecting 20 - 40% of patients with diabetes. Nowadays, it represents the leading cause of End-stage renal disease (ESRD) worldwide. The incidence of ESRD due to DKD continues to rise annually (1). Consistent and elevated urinary albumin excretion is a key characteristic of DKD and plays a crucial role in the early detection and evaluation of the risk of DKD advancement (2). The kidney plays an important role in the regulatory system for bone and mineral metabolism. Patients with diabetic kidney disease exhibit an increased risk of osteoporosis attributable to decreased renal 25-hydroxyvitamin D-1α hydroxylase activity, enhanced urinary excretion of vitamin D-binding protein (VDBP) resulted from increased permeability of the glomerular filtration membrane, and imbalance in calcium and phosphorus metabolism in serum and bone (3). Research has indicated that an increased risk of brittle bone fractures in DKD patients is associated with reduced bone mass and impaired bone quality (4).

Osteocalcin (OC) is a non-collagenous protein consisting of 46 to 50 amino acids. Osteoblasts generate it, primarily depositing it in the bone matrix, with a minor portion entering the bloodstream. Serum osteocalcin is a marker of bone formation, can reflect the activity of osteoblasts, and is also a specific and sensitive indicator of bone transformation and bone mineralization in the body (5). Recent studies have shown a notable inverse relationship between osteocalcin and urinary albumin - to - creatinine ratio (UACR) in diabetic patients, indicating that low osteocalcin levels are strongly linked to the onset and progression of DKD (6). And the recent prospective cohort study also confirmed that low osteocalcin levels are associated with an increased risk of DKD (7). Therefore, this study took Chinese patients with DKD as the research objects and conducted a systematic review and meta-analysis of the results of published case-control studies, aiming to explore the relationship between serum osteocalcin levels and DKD progression and provide new ideas and evidence-based medical evidence for the clinical prevention and treatment of bone metabolism abnormalities in patients with DKD.

## 2 Data and methods

This meta-analysis was conducted according to the guidelines of the Preferred Reporting Items for Systematic Reviews and Meta-Analyses (PRISMA). This study has been registered on PROSPERO (CRD42024580324).

### 2.1 Literature screening criteria

Inclusion criteria: (1) publicly published case-control studies; (2) According to Mogensen standard (8) and American Diabetes Association (ADA) standard (9), DKD is divided into three stages according to urinary albumin excretion rate (UAER) or UACR: simple diabetes mellitus (UAER < 30 mg/24 h, or UACR < 30 mg/g); microalbuminuria (30 ≤ UAER < 300 mg/24 h, or 30 ≤ UACR < 300 mg/g); macroalbuminuria (UAER ≥ 300 mg/24 h, or UACR ≥ 300 mg/g); (3) all cases are Chinese population; (4) no cases involve the use of medicines that impact bone metabolism or have any other conditions influencing bone metabolism; (5) the literature that was published multiple times within the same study is chosen based on having a large sample size.

Exclusion criteria: (1) repeated publication of literature; (2) unclear diagnostic criteria of the case; (3) patients have chronic kidney disease other than DKD or other diseases affecting bone metabolism; (4) patients need dialysis treatment; (5) patients use drugs that affect bone metabolism; (6) research data are incomplete or unable to extract valid data; (7) review, animal experiments or non-case-control studies.

### 2.2 Document retrieval strategy

PubMed, Web of Science, China Knowledge Network (CNKI), Wanfang Database, China Science and Technology Journal Database (VIP), and Chinese Medical Journal full-text Database were searched to collect the case-control studies related to serum osteocalcin and DKD from the establishment of the database to September 2024. The subject words combined with free words are used for retrieval. The search words include Chinese and English forms of osteocalcin, N-terminal osteocalcin, bone γ-carboxyglutamate protein, Diabetic Kidney Disease, Diabetic Nephropathy, and others.

### 2.3 Data extraction

All literature data were independently extracted by two researchers and then merged and proofread. Begin by removing duplicate literature. Next, a preliminary screening based on the inclusion and exclusion criteria will be conducted through titles and abstracts. Then, the full text of the selected literature will be reviewed to confirm if the study meets the criteria for inclusion in the analysis. If discrepancies arise during this time, the corresponding author will help make decisions. Data is collected by creating an Excel table that includes the first author, publication year, sample size, grouping standards, serum osteocalcin levels, and other relevant details.

### 2.4 Document quality evaluation

The study utilized the Newcastle-Ottawa scale (NOS) to assess bias risk. The scale consists of eight questions across three aspects: population selection, inter-group comparability, and measurement of exposure. It concretely consists of 4 entries on selection (adequacy of the case definition; representativeness of the cases; selection of Controls; definition of Controls), 1 entry on comparability (comparability of cases and controls on the basis of the design or analysis), and 3 entries on exposure (ascertainment of exposure; same method of ascertainment for cases and controls; non-response rate); all entries are worth a maximum of 1 point except for the one on comparability, which is worth a maximum of 2 points, with a maximum total score of 9 points. The higher total score show better quality of the study.

### 2.5 Statistical methods

Stata16 software was used for Meta-analysis. The study utilized standard mean difference (SMD) as the impact index for the continuous variables, with each effect being assigned a point estimate and a 95% confidence interval (CI). A difference was considered statistically significant if P ≤ 0.05. The heterogeneity between the results was included by Q test and I^2^ quantitative analysis. If P ≥ 0.1 and I^2^ ≤ 50%, the research results show no statistical difference in heterogeneity, and the fixed effect model is used for Meta analysis. If not, there are statistical differences in heterogeneity, and the random effect model is used for Meta analysis. Publication bias was assessed using a funnel plot, while sensitivity analysis was conducted to ensure the stability and reproducibility of the research findings. Potential publication bias was examined by Begg and Egger tests, with statistical significance set at P < 0.05.

## 3 Result

### 3.1 Results of literature retrieval

**Figure 1:**
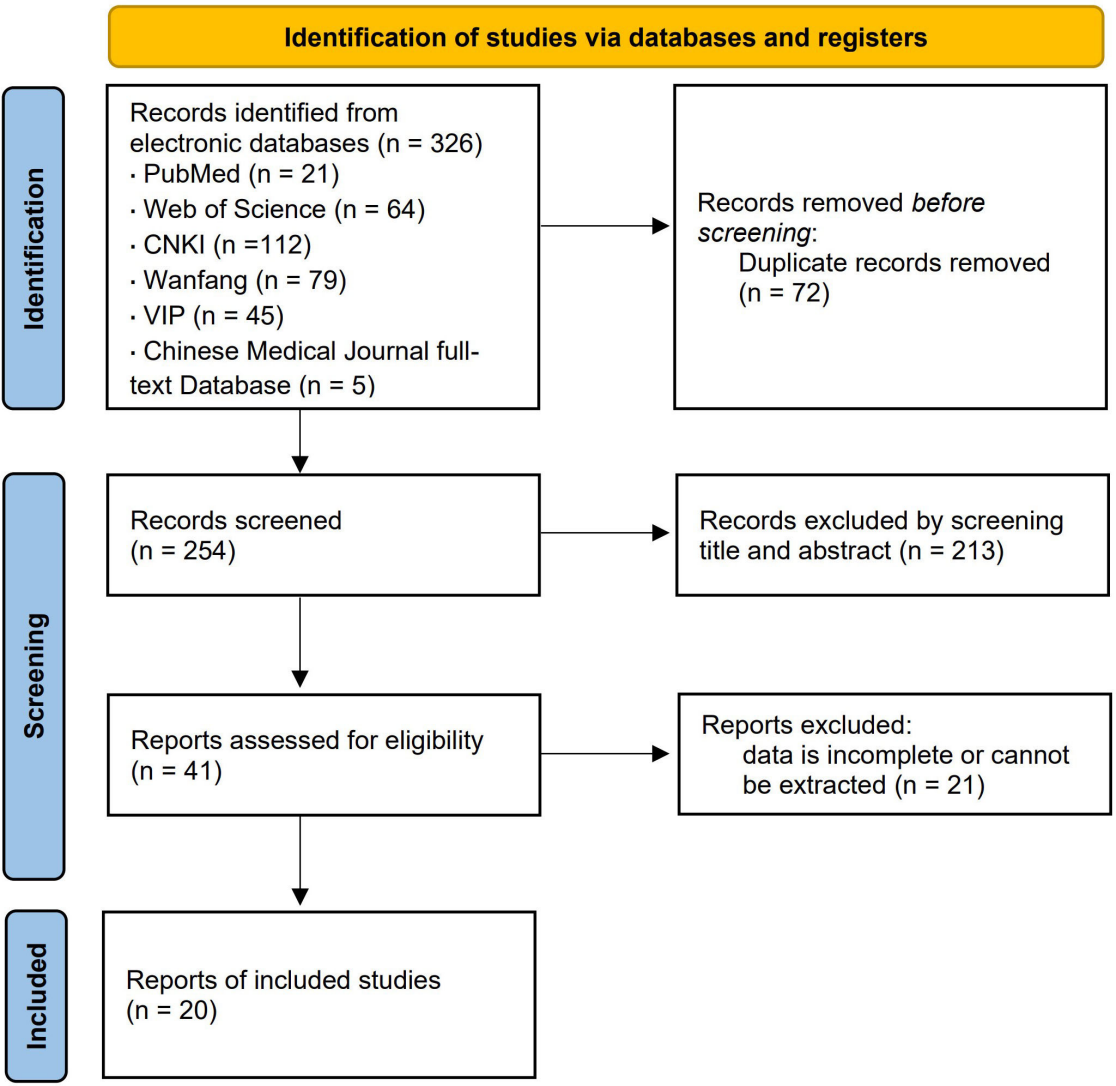
Flow chart of the Studies screening process.

A total of 326 related literature was found, and 254 articles were acquired following re-selection and primary screening. After reviewing the entire text, 20 case-control studies were analyzed, with a total sample size of 4 565 cases, distributed among healthy control (CN) group (n = 643), simple diabetes mellitus (DM) group (n = 1 649), microalbuminuria (MI) group (n = 1 305), and macroalbuminuria (MA) group (n = 968). The literature screening process and results are shown in **Figure 1**.

### 3.2 Basic characteristics and quality evaluation of included literature

A total of 20 studies were included, all of which were case-control studies, all of which were Chinese population, involving a total of 4565 samples. The Newcastle-Ottawa Scale is used to evaluate the quality of 20 included studies. Following a quality assessment, each piece of literature received a score ranging from 5 to 9, indicating a medium to high level of quality. The basic characteristics of the included study and the NOS score of the literature are shown in **Table 1**.

**Table 1:** Basic information and quality evaluation of the included research.

Study	Group	Sample size (n)	Age (years)	Course of DM (years)	OC (ng/ml)	NOS Score
Hu Z (1998) (10)	CN	60	49.8±8.6	0.0±0.0	5.48±1.53	8
	DM	165	51.3±13.8	6.6±6.4	4.35±1.84	
	MI	59	57.8±9.8		3.47±1.44	
	MA	20	58.1±12.7		3.30±1.52	
Gu B, (2010) (11)	CN	30	60-85	0.0±0.0	21.60±7.26	6
	DM	30		5-20	18.71±7.76	
	MI	30			14.04±4.61	
	MA	30			19.41±12.59	
Gao M, (2014) (12)	DM	51	62.4±8.3	11.3±6.7	15.12±7.37	9
	MI	40			12.99±4.81	
	MA	28			10.63±4.34	
Wang F, (2014) (13)	CN	30	48.3±9.7	0.0±0.0	16.05±5.68	7
	DM	92	48.4±9.0	6.6±6.0	13.68±5.82	
	MI	47	47.9±10.0	5.9±5.4	12.12±4.47	
	MA	21	50.5±10.2	7.6±5.1	14.26±8.52	
Chi H, (2015) (14)	DM	21	50.21±9.68	10.5±5.2	15.16±4.83	8
	MI	26		8.3±3.5	12.88±4.03	
	MA	18		12.5±3.8	10.92±3.94	
Qin K, (2016) (15)	DM	34	55.62±12.53		14.7±3.0	7
	MI	32	62.27±13.57		12.4±2.5	
	MA	32	59.9±10.99		9.0±1.4	
Mei L, (2016) (16)	CN	207	56.3±11.4		19.9±6.9	6
	DM	197	56.9±13.5		17.8±7.7	
	MI	205	57.2±14.7		15.3±7.5	
	MA	208	58.3±13.3		13.5±7.5	
Zhang L, (2017) (17)	DM	19	54.74±8.92	6.45±1.72	15.47±4.91	7
	MI	23		7.71±2.16	12.23±4.16	
	MA	25		9.64±3.82	10.81±3.49	
Miao C, (2017) (18)	DM	132		8.4±6.1	17.47±4.92	8
	MI	101		10.0±5.3	15.41±5.58	
	MA	69		12.9±6.2	14.39±3.75	
Fu T, (2018, Male) (19)	DM	60	60.86±9.90		14.70±17.12	8
	MI	38	60.49±12.54		17.12±14.88	
Fu T, (2018, Female) (19)	DM	56	63.66±9.08		18.95±8.28	8
	MI	33	63.88±10.66		16.54±7.79	
Zhou H, (2018) (20)	DM	37	52.17±6.81	12.53±3.87	16.32±5.15	6
	MI	39	51.36±5.77	8.63±2.72	14.09±4.53	
	MA	28	51.44±6.53	13.42±4.11	12.13±4.19	
Zhao X, (2019) (21)	DM	139	59.57±9.18	7.90±6.87	13.34±5.30	9
	MI	94	60.79±14.72	10.41±7.68	13.67±5.89	
	MA	64	63.50±12.20	15.75±6.88	20.78±12.70	
Ren H, (2019, Male) (6)	CN	141	54.09±10.79		18.01±6.77	8
	DM	138	54.05±10.69		15.73±5.05	
	MI	127	52.17±9.82		12.24±3.03	
	MA	109	55.19±9.02		10.14±1.8	
Ren H, (2019, Female) (6)	CN	139	64.68±10.53		26.55±9.03	8
	DM	135	64.71±10.56		21.26±8.07	
	MI	125	63.46±9.96		17.89±5.90	
	MA	104	62.05±11.34		13.10±5.01	
Zhao W, (2019) (22)	DM	29	64.84±11.3	9.69±6.69	16.89±8.91	7
	MI	28		13.66±7.32	11.65±11.29	
	MA	22		17.41±8.76	10.27±23.83	
Li J, (2021) (23)	DM	36	52.3±6.8	12.5±3.5	16.3±5.0	5
	MI	40	51.7±5.9	8.6±2.7	14.1±4.6	
	MA	30	51.5±6.0	13.4±4.0	12.2±4.1	
Lu Y, (2021) (24)	DM	38	57.21±8.24	7.76±5.04	18.67±3.72	7
	MI	37	58.60±12.2	7.83±6.06	15.70±5.16	
	MA	12	61.60±13.8	10.83±2.55	11.64±5.07	
Guo M, (2021) (25)	DM	73	65.96±6.50	14.82±7.77	14.21 ± 5.62	6
	MI	30	67.60±8.30	13.60±7.44	11.90 ± 4.22	
	MA	28	66.93±7.13	14.43±7.95	9.80 ± 3.25	
Liu J, (2022) (26)	DM	80	62.41±7.49	6.64±1.39	12.46±2.11	8
	MI	62	63.10±6.74	8.32±2.22	9.30±1.69	
	MA	40	62.88±7.51	11.58±2.74	7.83±1.08	
Liu Y, (2022) (27)	DM	47	52.16±6.80		16.31±5.16	6
	MI	49	51.38±5.76		14.10±4.55	
	MA	38	51.45±6.54		12.14±4.20	
Han L, (2023) (28)	CN	36			32.05±9.52	8
	DM	40			24.22±7.46	
	MI	40			15.36±5.84	
	MA	42			9.91±5.19	

NOS, Newcastle-Ottawa scale; CN, healthy control; DM, simple diabetes; MI, diabetic microalbuminuria; MA, diabetic macroalbuminuria; N-terminal osteocalcin (N-MID); osteocalcin (OC, BGP).

### 3.3 Results of meta-analysis

#### 3.3.1 Comparison of serum osteocalcin between MI group and CN group

**Figure 2:**
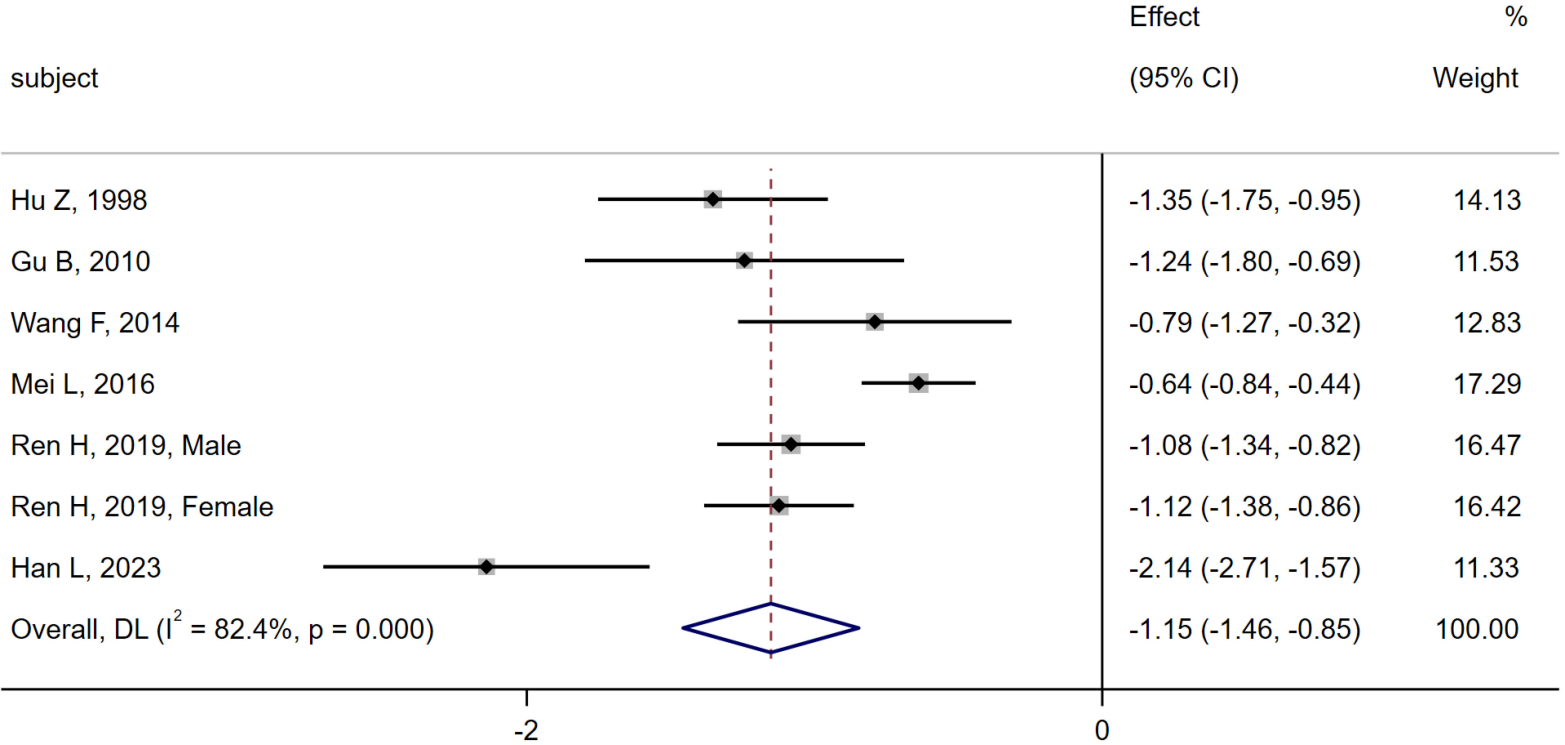
Meta-analysis forest plot comparing serum osteocalcin levels between MI group and CN group.

6 studies (6, 10, 11, 13, 16, 28) were compared between the MI group (n = 633) and the CN group (n = 643). Heterogeneity analysis showed Q = 34.05, I^2^ = 82.4%, P < 0.01, and the random effect model was applied. The meta-analysis results indicated a significant decrease in serum osteocalcin levels in the MI group compared to the CN group [SMD = -1.15, 95% CI (-1.46, -0.85), P < 0.01]. As shown in **Figure 2**.

#### 3.3.2 Comparison of serum osteocalcin between MI group and DM group

**Figure 3:**
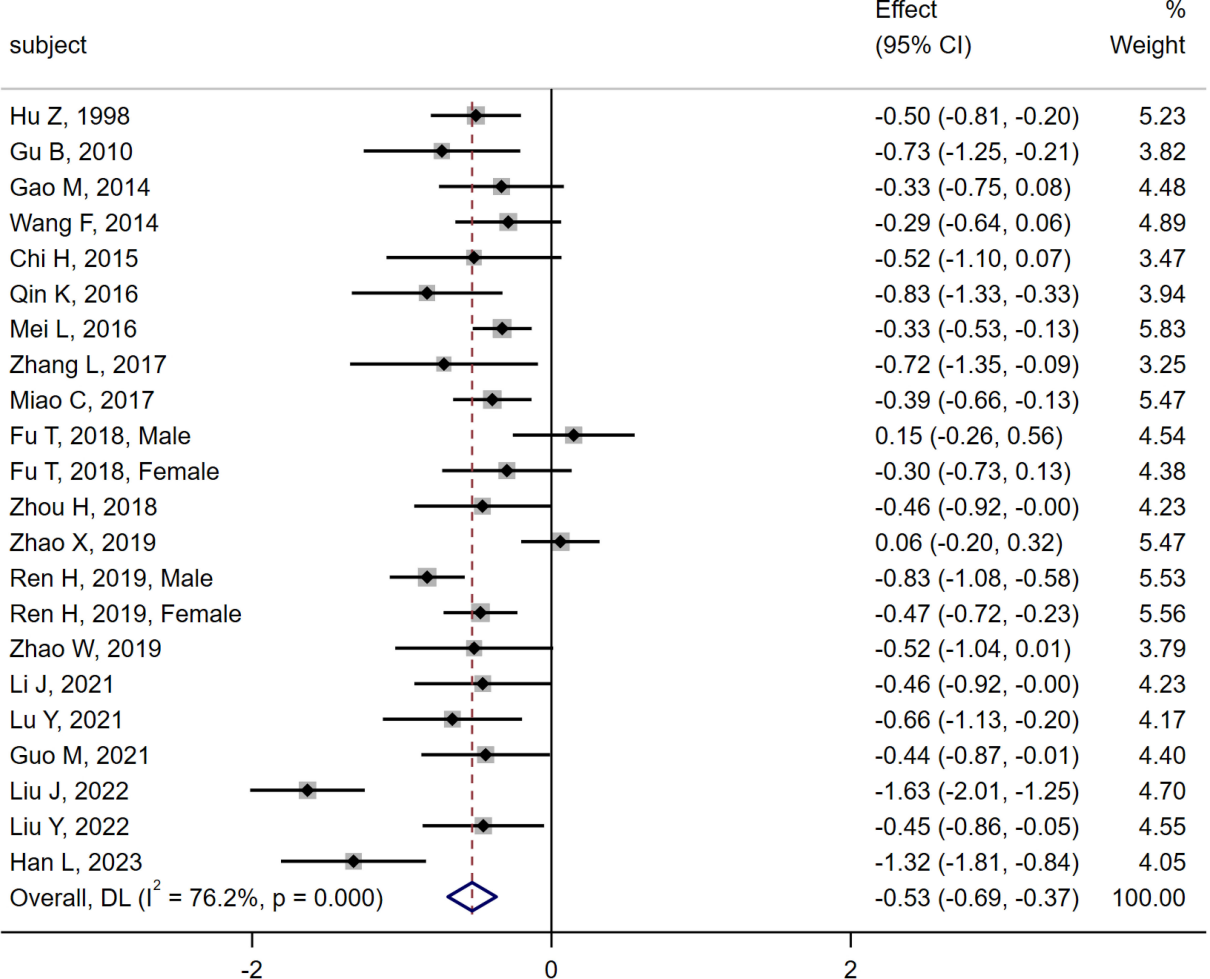
Meta-analysis forest plot comparing serum osteocalcin levels between MI group and DM group.

20 studies (6, 10–28) were compared between the MI group (n = 1 305) and the DM group (n = 1 649). Heterogeneity analysis indicated Q = 88.21, I^2^ = 76.2%, P < 0.01, and the random effect model was used for analysis. The meta-analysis results showed that the serum osteocalcin level in the MI group was significantly lower than that in the DM group [SMD = -0.53, 95% CI (-0.69, -0.37), P < 0.01]. As shown in **Figure 3**.

#### 3.3.3 Comparison of serum osteocalcin between MA group and CN Group

**Figure 4:**
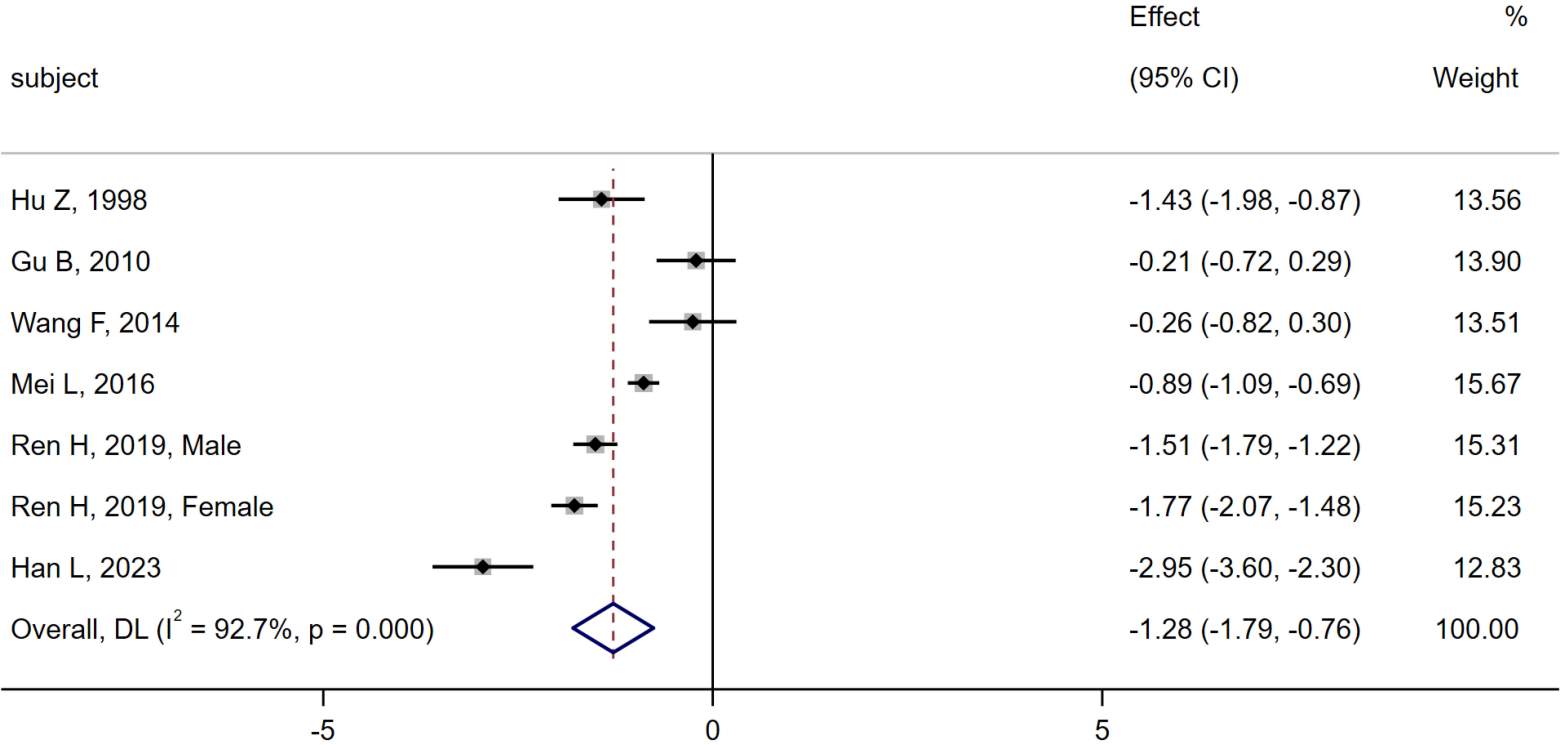
Meta-analysis forest plot comparing serum osteocalcin levels between MA group and CN group.

6 studies (6, 10, 11, 13, 16, 28) were compared between the MA group (n = 534) and the CN group (n = 643). Heterogeneity analysis indicated Q=82.08, I^2^ = 92.7%, P < 0.01, thus the random effect model was used. The meta-analysis showed a significant decrease in serum osteocalcin levels in the MA group compared to the CN group [SMD = -1.28, 95% CI (-1.79, -0.76), P < 0.01]. As shown in **Figure 4**.

#### 3.3.4 Comparison of serum osteocalcin between MA group and DM group

**Figure 5:**
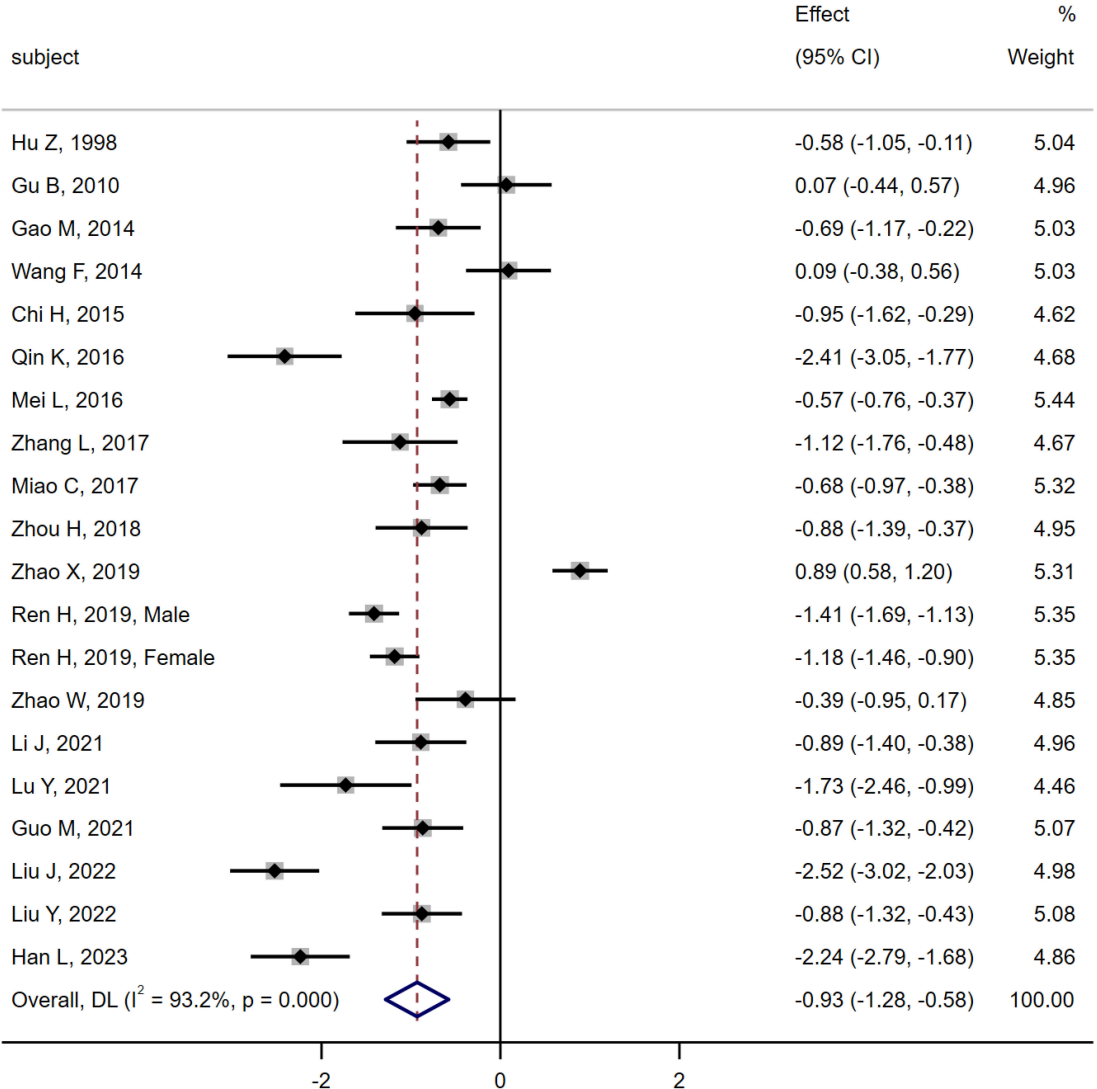
Meta-analysis forest plot comparing serum osteocalcin levels between MA group and DM group.

19 studies (6, 10–18, 20–28) were compared between the MA group (n = 968) and the DM group (n = 1 533). Heterogeneity analysis indicated Q = 278.45, I^2^ = 93.2%, P < 0.01, so the random effect model was used. The results of the meta-analysis showed that the level of serum osteocalcin in the MA group was significantly lower than that in the DM group [SMD = -0.93, 95% CI (-1.28, -0.58), P < 0.01]. As shown in **Figure 5**.

#### 3.3.5 Comparison of serum osteocalcin between MA group and MI group

**Figure 6:**
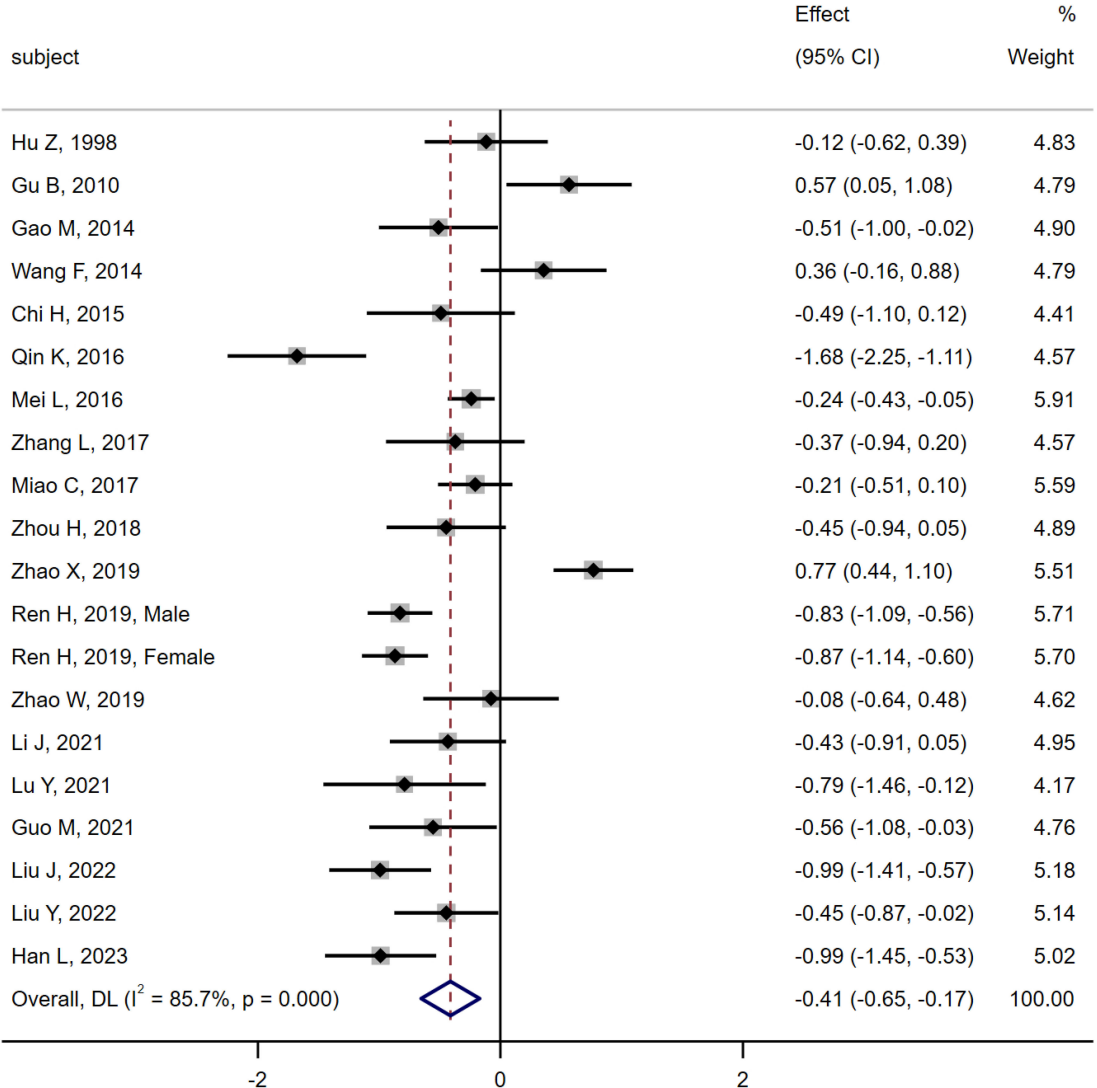
Meta-analysis forest plot comparing serum osteocalcin levels between MA group and MI group.

19 studies (6, 10–18, 20–28) were compared between the MA group (n = 968) and the DM group (n = 1 234). The results of heterogeneity analysis were as follows Q =133.04, I^2^ = 85.7%, P < 0.01, using random effect model. The results of the meta-analysis showed that the level of serum osteocalcin in the MA group was significantly lower than that in the MI group [SMD = -0.41, 95% CI (-0.65, -0.17), P < 0.01]. As shown in **Figure 6**.

### 3.4 Sensitivity analysis

**Figure 7:**
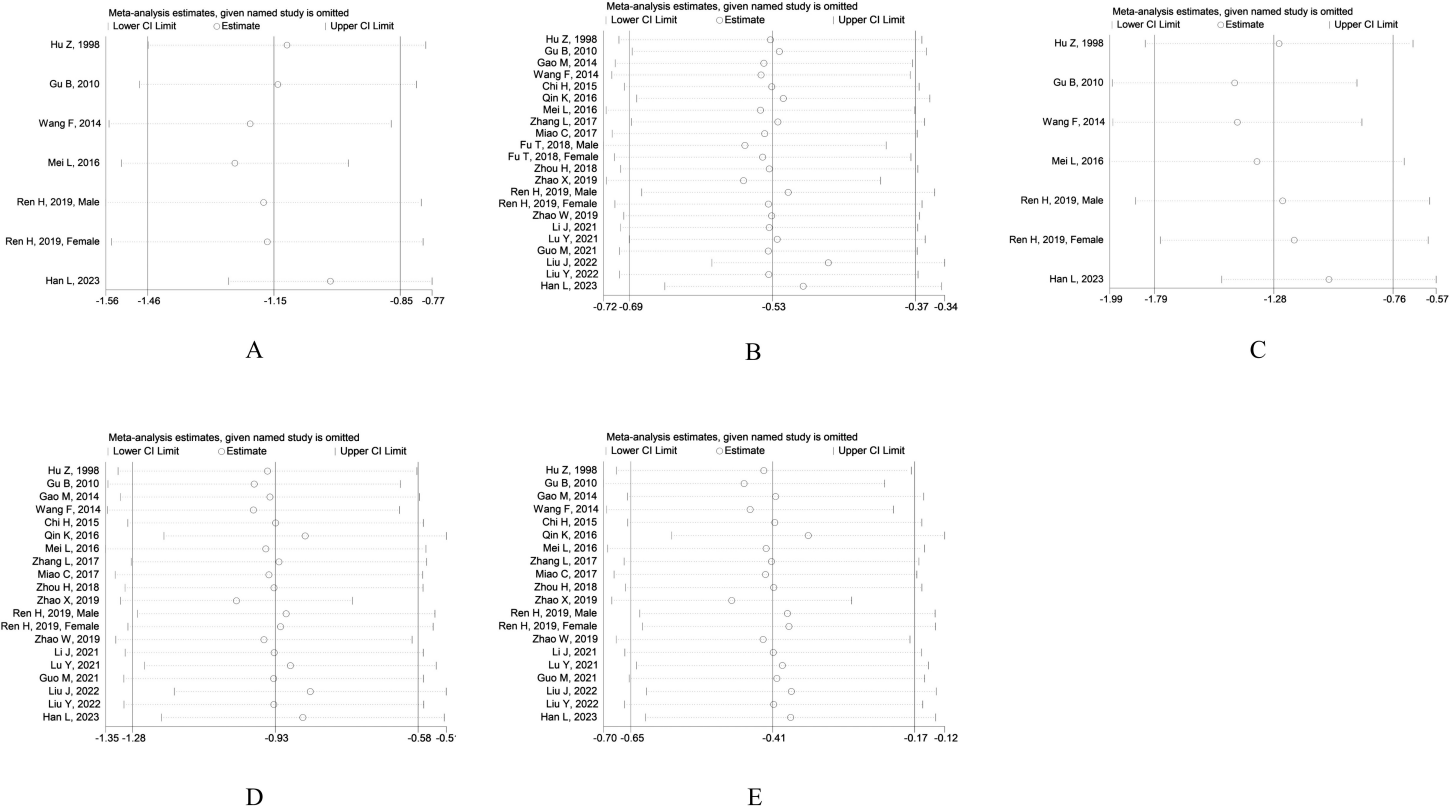
Sensitivity analysis. **(A)** Sensitivity analysis of MI group and CN group; **(B)** Sensitivity analysis of MI group and DM group; **(C)** Sensitivity analysis of MA group and CN group; **(D)** Sensitivity analysis of MA group and DM group; **(E)** Sensitivity analysis of MA group and MI group.

The sensitivity of each group included in the study was analyzed by excluding a single research one by one. The study results showed that the combined SMD value and the 95% CI were not significantly changed before and after exclusion in all study groups, with no statistically significant difference appearing. The preceding meta-analysis is robust and the findings are dependable (**Figure 7**).

### 3.5 Publication bias

**Figure 8:**
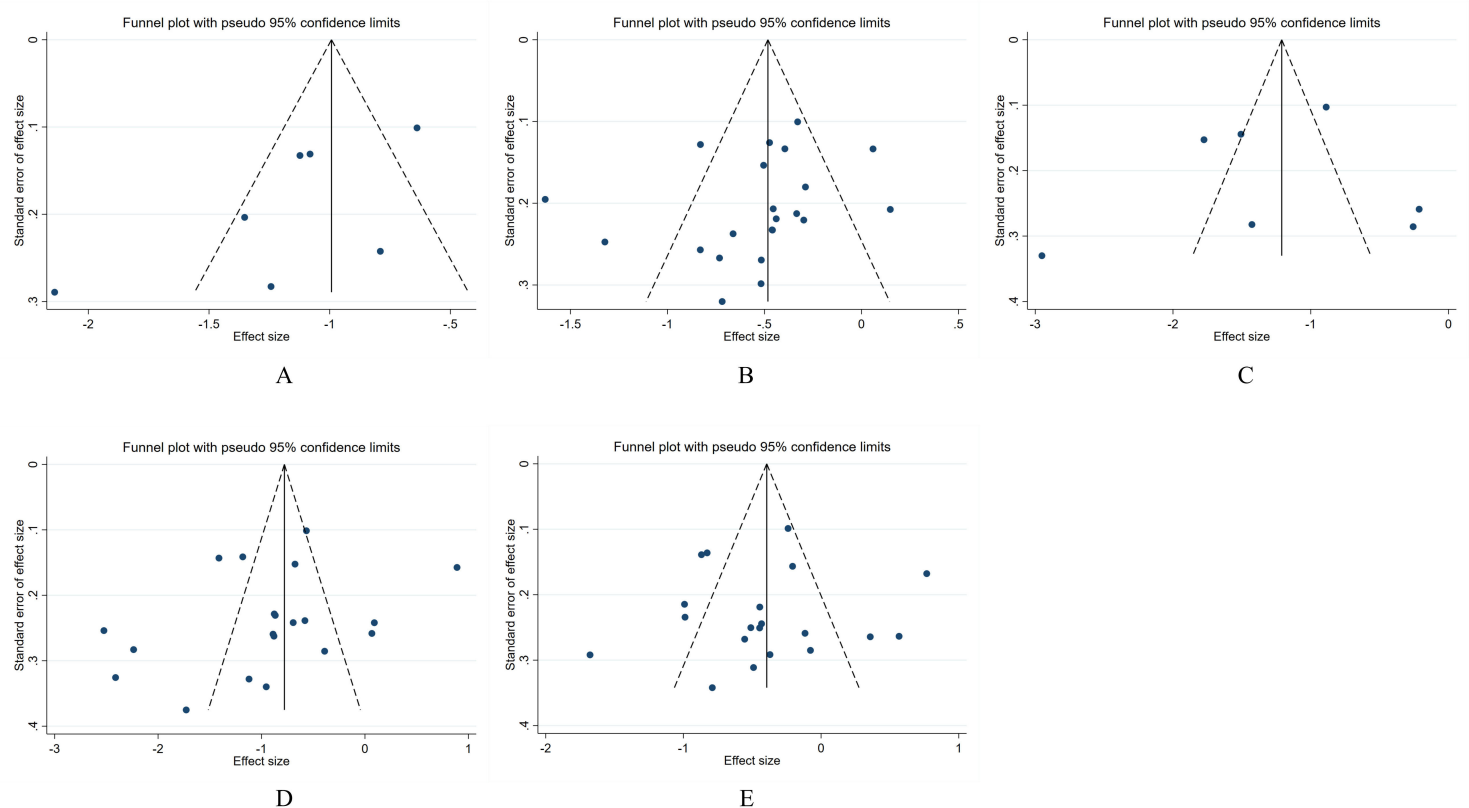
Publication bias of the relationship between serum osteocalcin levels and the progression of DKD in the Chinese population. **(A)** Analysis of publication bias between MI group and CN group; **(B)** Analysis of publication bias between MI group and DM group; **(C)** Analysis of publication bias between MA group and CN group; **(D)** Analysis of publication bias between MA group and DM group; **(E)** Analysis of publication bias between MA group and MI group.

Publication bias was assessed by drawing funnel plots, which showed some graphical asymmetry (**Figure 8**), indicating publication bias. The reasons may be related to the poor quality of some literature, the complexity of the sample population, and the existence of unpublished negative results. The Begg test and Egger test results showed no statistical difference in the potential publication bias analysis of the combined effect size of the included groups. The details are as follows: MI group vs CN group: Begg’s test, P = 0.133, Egger’s test, P = 0.081; MI group vs DM group: Begg’s test, P=0.063, Egger’s test, P=0.236; MA group vs CN group: Begg’s test, P=0.368, Egger’s test, P=0.745; MA group vs DM group: Begg’s test, P=0.256, Egger’s test, P=0.251; MA group vs MI group: Begg’s test, P=0.974, Egger’s test, P=0.823.

## 4 Discussion

The prevalence of osteoporosis in China is increasing quickly due to the aging population. The survey found that osteoporosis affects 19.2% of individuals over 50 years old in China and 32.0% of those over 65 years old, with women accounting for 51.6% of cases (29). And studies have shown that patients with diabetes and/or chronic kidney disease have a higher risk of osteoporosis and brittle fractures than the general population (30, 31). DKD is not only a common microvascular complication of diabetes, but also the main cause of chronic kidney disease and ESRD (2). Therefore, the DKD population should pay more attention to the screening and prevention of osteoporosis to avoid fractures.

Osteoporosis has no noticeable symptoms in the early stages, but abnormal bone metabolism occurs. Many patients are not detected and diagnosed until fractures occur, which often places a heavy burden on individuals, families, and society. Dual energy X-ray absorptiometry (DXA) is currently the preferred method for diagnosing osteoporosis in clinical settings. However, it has limits in early diagnosis, predication of fracture risk and evaluation of therapeutic effect. On the contrary, although bone metabolism indicators are not suitable for diagnosing osteoporosis, they can reflect early bone metabolism abnormalities due to their high sensitivity and specificity (32), thus are functional in differential diagnosis, risk prediction, bone turnover type judgment, and treatment evaluation of osteoporosis (33), and are widely used in clinical practice.

Osteocalcin, encoded by the bone γ-carboxyglutamate protein gene, is the only non-collagenous protein secreted by osteoblasts during osteogenesis, and the expression of its biological activity is vitamin K-dependent (34). Serum osteocalcin is relatively stable and can directly reflect the status of bone formation, so it has been used as a clinical indicator of bone formation markers (33). Interestingly, new studies have found that osteocalcin, although secreted by osteoblasts during bone formation, is released into the circulation during bone resorption, and is therefore also recognized as a bone turnover marker (35).

This meta-analysis indicates that the serum osteocalcin levels in Chinese patients with DKD are significantly lower than in healthy population and patients with simple DM. With the increase of the diabetes course and the severity of proteinuria in DKD patients, the serum osteocalcin level exhibits a gradual decline, indicating that patients with DKD are characterized by a decrease in osteoblast activity at the early stage and low conversion type of bone turnover.

**Figure 9:**
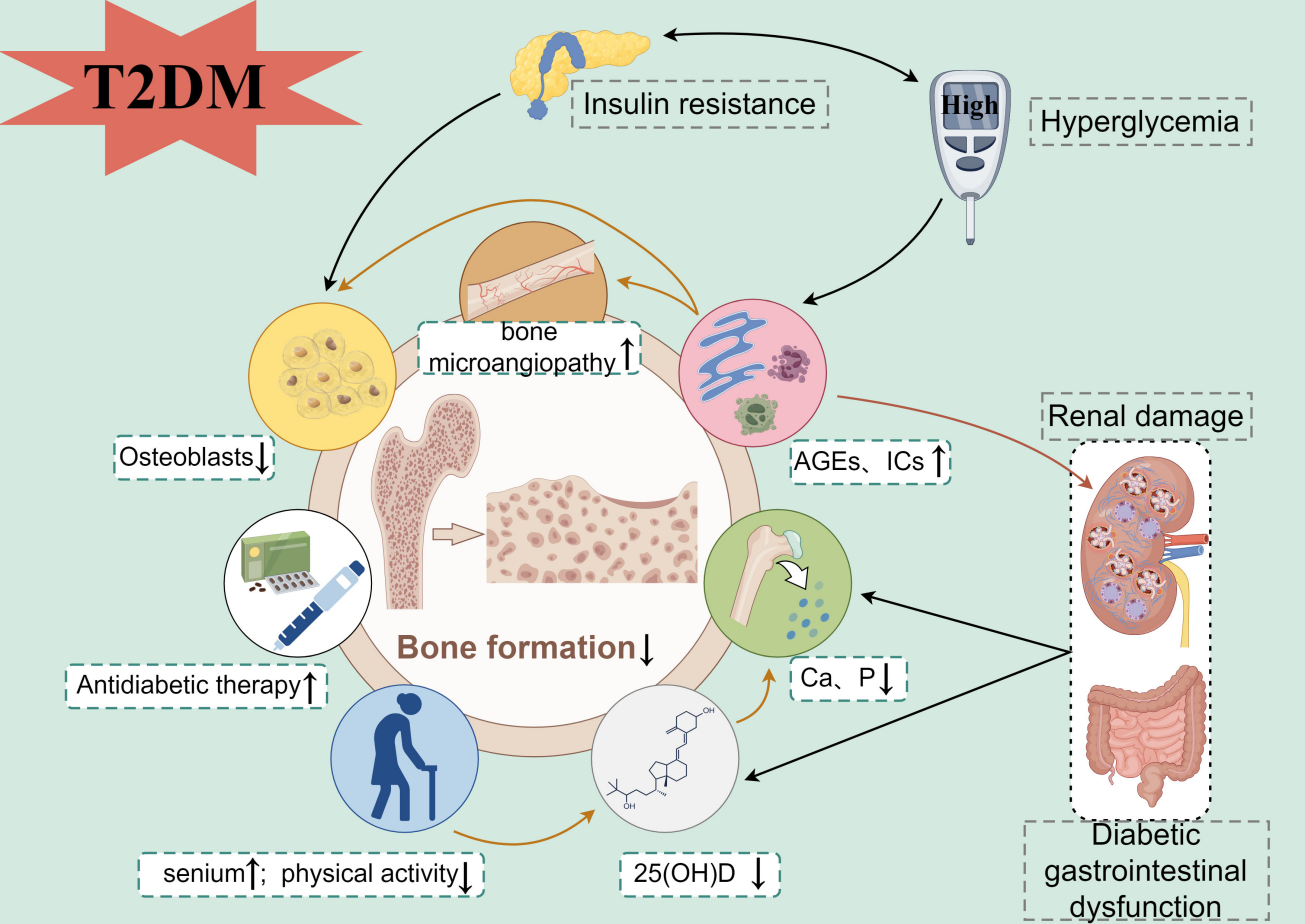
Mechanism map of bone formation reduction in patients with DKD (By Figdraw). T2DM, type 2 diabetes mellitus; AGEs, advanced glycation end products; ICs, pro-inflammatory cytokines; Ca, Calcium; P, Phosphorus; 25(OH)D, 25-hydroxyvitamin D.

The mechanism of reduced bone formation in patients with DKD has not been fully elucidated and may be related to the following factors. First, renal damage: In patients with DKD, there is impaired renal filtration and reabsorption functions leading to increased loss of calcium, phosphorus, and various vitamins from the kidney. This results in decreased raw materials for bone formation, reduced function and activity of osteoblasts, and an imbalance between bone formation and resorption, ultimately causing osteoporosis (36). In addition, DKD patients can indirectly stimulate parathyroid hormone secretion, causing secondary hyperparathyroidism, which leads to increased osteoclast activity and decreased bone mineralization (37), further exacerbating the bone conversion imbalance. Second, 25-hydroxyvitamin D deficiency: The decrease of serum 25-hydroxyvitamin D level was common in patients with DKD, and the degree of decrease was positively correlated with the progress of DKD (38). 25-hydroxyvitamin D deficiency can promote the dysfunction of calcium and phosphorus reabsorption in renal tubules, inhibit osteocalcin synthesis by osteoblasts, decrease bone mineralization, and increase the risk of osteoporosis (36). Third, hyperglycemia: In DKD patients, insulin deficiency or resistance leads to poor control of blood glucose and disorder of glucose and lipid metabolism, which can lead to the accumulation of pathological products such as advanced glycation end products (AGEs) (39) and proinflammatory cytokines (ICs) (40), thus reducing bone formation and osteoblast activity and increasing bone resorption and osteoclast activity. And other studies have shown that chronic hyperglycemia can reduce osteocalcin gene transcription and osteoblast differentiation at the gene transcription level (41, 42). In addition, long-term hyperglycemia will also cause microvascular lesions in the body. when bone microvascular lesions occur, the bone blood supply is insufficient and bone metabolism is out of balance, resulting in the destruction of bone microstructure and the decrease of bone formation and bone mineralization level (43). Fourth, insulin resistance: Insulin at a biological dose can enhance osteoblast proliferation, differentiation, and activity while reducing osteoclast activity, but in cases of insulin resistance, there is a disruption in bone energy metabolism leading to increased bone matrix consumption, decreased bone formation, reduced bone turnover, and increased bone fragility (44). Fifth, diabetic gastrointestinal dysfunction: Patients with DKD have been experiencing diabetes for an extended period, leading to complications such as gastrointestinal dysfunction. Due to reduced gastrointestinal absorption function and dietary restrictions imposed by diabetes, the intake of calcium, phosphorus, and vitamins may not suffice to support normal bone formation, resulting in decreased bone formation (45). Sixth, antidiabetic therapy: Cipriani and his colleagues reviewed the published studies and found that long-term use of antidiabetic drugs such as insulin and thiazolidinediones can have a negative impact on bone health (46), which is one of the risk factors for fracture and osteoporosis. Seventh, senium: Human serum osteocalcin levels peak in young individuals and subsequently drop with aging and declining exercise ability (47, 48). Correspondingly, the proliferation and differentiation of osteoblasts decreased during senescence, and the physiological function reduced, resulting in a decrease in the level of bone formation. Furthermore, the outdoor activities of the elderly decreased, the synthesis of 25-hydroxyvitamin D decreased, and the effect of bone stress was weakened, which further promoted the reduction of bone formation. In summary, DKD is a chronic microvascular complication of diabetes, and most of its patients are elderly and have a long course of disease. The decrease in bone formation in patients with DKD may be the result of various pathological conditions caused by prolonged diabetes and aging (**Figure 9**).

Interestingly, the four included studies (11, 13, 19, 21) showed that patients in the MA group had higher osteocalcin levels than those in the MI or DM group, inconsistent with the results obtained from the meta-analysis. The reasons may be related to the changed lifestyle and the decline in the different degrees of renal function in the macroalbuminuria population. Patients with macroalbuminuria have more obvious clinical symptoms and abnormal test indicators, which may force them to pay more attention to lifestyle and physical activity, affecting the original bone turnover. Furthermore, patients with macroalbuminuria or renal failure have significantly increased renal function impairment, the role of renal tubules gradually changes from compensatory increase to decrease, and secondary hyperparathyroidism results in pathological enhancement of both osteoblast and osteoclast activities (49). At this time, the transformation of osteoclast to osteoblast is accelerated, serum osteocalcin levels also increase, and bone turnover changes from a low turnover type in early DKD to a high one. It shows that bone metabolism in DKD patients may have inconsistent characteristics at different stages of renal damage.

The limitations of this study are as follows. First, some included studies have small sample sizes, particularly for MA groups, which may reduce the reliability of the meta-analysis. Second, the methodological quality of this study is relatively low. Despite rigorous screening of included studies according to uniform criteria, there is still biggish heterogeneity in the meta-analysis, which may be related to differences in study design, sample population selection, and measurement methods of OC among included studies. Third, this study only included the Chinese population, and the lack of diversity in population selection may limit the universality of the results to other ethnic groups with different genetic backgrounds and regions. Forth, due to the complexity of the sample population, the study could not exclude confounding factors such as diet, physical activity, and other comorbidities. Finally, the funnel plots show that this study has publication bias, which may affect the accurate judgment of the association between osteocalcin and DKD. Therefore, to verify this study, more clinical studies with rigorous design, authentic records, and large sample sizes are needed. Recommending that future research on serum osteocalcin levels and the progression of DKD should focus more on improving methodological quality, clearly recording the characteristics of the study population, unifying serum osteocalcin measurement methods, and expanding the sample size and the representativeness of the study population, to provide better evidence-based medical evidence for subsequent research and clinical practice.

## 5 Conclusion

This research indicates that the decrease in serum osteocalcin is prevalent among Chinese patients with DKD. This reduction is strongly related to the severity of proteinuria, indicating a decline in bone formation at the early stages of DKD that worsens as the disease progresses. However, due to the limitations of the quantity and quality of the included studies, the above conclusions need to be verified by more high-quality, large-sample clinical studies.

## Data Availability

The original contributions presented in the study are included in the article/supplementary material. Further inquiries can be directed to the corresponding authors.
